# Correction: Reproductive health programs in women with physical disabilities: A scoping review protocol

**DOI:** 10.1371/journal.pone.0336878

**Published:** 2025-11-14

**Authors:** Maryam Abedi, Mehran Soleymani, Seyedeh Solmaz Talebi, Zahra Motaghi

The Acknowledgements statement for this article is missing: This study was part of a PhD thesis approved by Shahroud University of Medical Sciences (thesis code: 1469) and was approved by its Ethics Committee (Ref. ID: IR.SHMU.REC.1402.053). We thank the Research Deputy of Shahroud University of Medical Sciences, Shahroud, Iran for their support.
