# Supplementary material for: Reproductive health programs in women with physical disabilities: A scoping review protocol
Source: PLoS One. 2025 Aug 6;20(8):e0329124. doi: 10.1371/journal.pone.0329124 (PMC12327595; doi:10.1371/journal.pone.0329124)
Supplement: S1 Table — (PDF) [file pone.0329124.s003.pdf]

| **Keyword search** | **Date of search/ Search engine used** | **Number of publications retrieved** |
|---|---|---|
| **"((((((((Reproductive Health) AND (program)) OR (guideline)) OR (promotion program)) AND (""Physical disability"")) OR (""physical limitations"")) OR (""Physical-Motor Disabilities"")",,"Full text, Humans, English, Persian, Female, from 2006 - 2024","(((((""reproductive health""[MeSH Terms] OR (""reproductive""[All Fields] AND ""health""[All Fields]) OR ""reproductive health""[All Fields]) AND (""program""[All Fields] OR ""program s""[All Fields] OR ""programe""[All Fields] OR ""programed""[All Fields] OR ""programes""[All Fields] OR ""programing""[All Fields] OR ""programmability""[All Fields] OR ""programmable""[All Fields] OR ""programmably""[All Fields] OR ""programme""[All Fields] OR ""programme s""[All Fields] OR ""programmed""[All Fields] OR ""programmer""[All Fields] OR ""programmer s""[All Fields] OR ""programmers""[All Fields] OR ""programmes""[All Fields] OR ""programming""[All Fields] OR ""programmings""[All Fields] OR ""programs""[All Fields])) OR (""guideline""[Publication Type] OR ""guidelines as topic""[MeSH Terms] OR ""guideline""[All Fields]) OR ((""promote""[All Fields] OR ""promoted""[All Fields] OR ""promotes""[All Fields] OR ""promoting""[All Fields] OR ""promotion""[All Fields] OR ""promotional""[All Fields] OR ""promotions""[All Fields] OR ""promotive""[All Fields]) AND ""Physical disability""[All Fields]) OR ""physical limitations""[All Fields] OR ""Physical-Motor Disabilities""[All Fields]) AND ((fft[Filter]) AND (humans[Filter]) AND (female[Filter]) AND (English[Filter] OR Persian[Filter]) AND (2006:2024[pdat]))",** | **31jan2024/ MEDLINE via PubMed** | **843** |
