# Supplementary figures and images for: Reproductive health programs in women with physical disabilities: A scoping review protocol
Source: PLoS One. 2025 Aug 6;20(8):e0329124. doi: 10.1371/journal.pone.0329124 (PMC12327595; doi:10.1371/journal.pone.0329124)

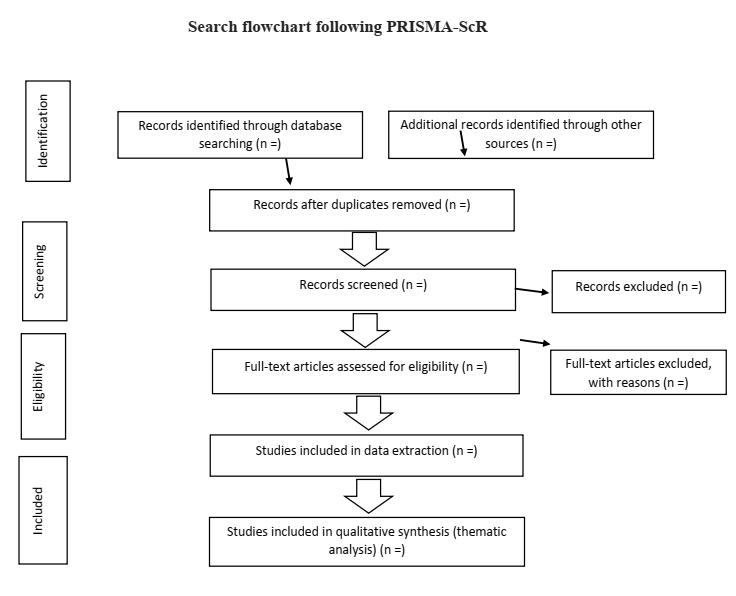

Supplement: S1 Fig — (TIF) [file pone.0329124.s004.tif]
