# Supplementary material for: Reproductive health programs in women with physical disabilities: A scoping review protocol
Source: PLoS One. 2025 Aug 6;20(8):e0329124. doi: 10.1371/journal.pone.0329124 (PMC12327595; doi:10.1371/journal.pone.0329124)
Supplement: Attachment — Submitted filename: Response to Reviewers.docx [file pone.0329124.s005.docx]

**Subject:** Response to Reviewers' Comments on the Manuscript titled " **Reproductive health programs in women with physical disabilities: a scoping review protocol"**

**Dear Editor and Reviewers,**

Thank you very much for your time and effort in reviewing our manuscript titled "**Reproductive health programs in women with physical disabilities: a scoping review protocol"** and for providing us with constructive feedback. We greatly appreciate the valuable comments and suggestions, which have helped us improve the quality of our work.

We have carefully addressed all the reviewers' comments and made the necessary revisions to the manuscript. Below, we provide a point-by-point response to each comment and detail the changes made in the revised manuscript.

|  | Reviewer #1 |
| --- | --- |
| 1 | The very limited search terms the authors plan to use as in line 122. These search terms are very limited. Other terms used for these should be considered e.g impairment, activity limitation, participation limitation, sexual health etc. |
| answer | Thank you for your valuable feedback regarding the search terms. Based on your comments, we have added the following keywords to our search strategy Reproductive and sexual health, physical limitations, decreased mobility, immobility, Physical-Motor Handicap, impairment, activity limitation, Physical-Motor Disabilities, and program, care. These terms have been included in lines 151-153 of the revised manuscript. |
| 2 | Authors do not intend to do any form of quality assessment. I do not understand why they chose not to do this. A quality assessment is necessary if the authors intend making conclusions, as they have done, on what are appropriate interventions and programs for women with disabilities |
|  | Thank you for your insightful comment. As the purpose of this scoping review is to map existing programs related to reproductive health for women with disabilities, our primary objective is not to draw conclusions or identify effective programs. However, we fully agree with your suggestion that assessing the included studies' quality can significantly improve our review's transparency and rigor, and provide an overview of the quality of existing studies and help identify research gaps. In response to your constructive input, we have incorporated a quality assessment step after the screening process and will provide an overview of the quality of existing studies. However, as stated, our goal in assessing the quality of studies is not to draw conclusions or select studies based on quality or to identify effective studies.  We sincerely appreciate your thoughtful suggestion, which has undoubtedly strengthened the quality of our work.  The description of the quality assessment process has been added to the Methods section (see lines 224-228). |
| 3 | Authors make very bold conclusions about the implications of their findings such as what are appropriate programs and interventions for women with disabilities. Given the two issues above, such a conclusion cannot be supported. Fundamentally, scoping reviews not ideal for designing programs or interventions. Their primary purpose is to scope the literature to identify gaps in the knowledge, determine feasibility of a systematic review and make recommendations on future research**.** |
|  | Thank you for your careful observation and valuable feedback. We also completely agree with your opinion, and our goal in this scoping review is to identify and summarize studies in the field of reproductive health programs for women with physical disabilities, and we are not looking for conclusions and identification of effective programs. In response to your comment, we have revised the Conclusion section to better align with the goals of a scoping review (see line38-44) |
|  | Finally, the manuscript can benefit from a through proofreading or making use of the services of a language editor. It is full of grammatical and typographical errors |
|  | Thank you for your valuable feedback. In response to your suggestion, we have carefully reviewed the entire manuscript and utilized the services of a professional language editor to address grammatical and typographical errors.  Please review the revised version, and if further edits are needed, we are happy to make additional adjustments. |

|  | Reviewer #2 |
| --- | --- |
| 1 | Please rewrite your abstract into subcategories for better clarity. |
| answer | Thank you for your valuable suggestion to improve the clarity of the abstract. We have rewritten the abstract and divided it into subsections to make its structure clearer and more readable (please see line 20-44). |
| 2 | Emphasize the type of study you are conducting. |
|  | Thank you for your valuable feedback. We have revised the manuscript in various sections, such as the introduction and discussion sections, to more clearly emphasize the type of study being conducted) please see line99-102,285-290). |
| 3 | I recommend replacing the term "disabled" with "individuals with special needs". |
|  | Thank you for your thoughtful suggestion regarding the terminology used in our manuscript. We appreciate your perspective on replacing the term "disabled" with "individuals with special needs." While we acknowledge the importance of using inclusive and respectful language, we have chosen to retain the term "disabled" for the following reasons:  It is widely recognized and used in scientific, legal, and policy contexts.  It carries specific social and cultural significance, particularly within the disability rights movement, where many individuals and organizations prefer this term as it emphasizes social barriers rather than focusing solely on individual limitations.  If you have specific suggestions for alternative terms or phrases in other parts of the manuscript, we would be happy to consider them. |
| 4 | In the introduction, clearly highlight the research gap that your study aims to address |
|  | Thank you for your feedback. We have revised the Introduction by adding sample studies and further explanation to better highlight the research gap (please see lines 61-100).  We appreciate your time and welcome any further suggestions. |

|  | Reviewer #3 |
| --- | --- |
| 1 | This would be good to resubmit when the project is complete and recommendations/ findings are included. |
| answer | Thank you for your valuable suggestion. We fully agree with your opinion that providing recommendations and findings after the project is completed can help improve the quality and make the study more applicable. Currently, this article is presented as a protocol, but after the project is completed and the data is analyzed, we will submit the final version with full findings and recommendations for re-review.  Thank you for your support and guidance. |

|  | Reviewer #4 |
| --- | --- |
| 1 | Lack of specificity: The abstract mentions the aim of the scoping review but does not clearly specify the types of reproductive health programs or interventions that will be explored. It could benefit from a more detailed overview of the expected findings. |
| answer | Thank you for your insightful feedback. In this scoping review, we aim to comprehensively identify reproductive health programs specifically designed for women with disabilities. This will include a wide range of interventions, such as Educational and awareness programs, Initiatives to improve access to healthcare services, support groups and advocacy efforts, and Policy-level interventions. These interventions will be explored across various dimensions of reproductive health.  This content has been added to the abstract section (please see lines 38-41). |
| 2 | Limited context: The introduction highlights the neglect of reproductive health needs for women with physical disabilities but lacks specific examples of existing programs or studies illustrating the current state of research in this area |
|  | Thank you for your valuable feedback. We fully agree with your comment that providing specific examples of existing programs or studies can help to better understand the current state of research in this area. In response to this suggestion, we have revised the Introduction section and added examples of programs and studies relevant to the reproductive health of women with physical disabilities (please see lines 61-91). |
| 3 | Insufficient justification: The rationale for the review’s importance could be strengthened by including statistics or data emphasizing the impact of inadequate reproductive health services on this population |
|  | Thank you for your feedback. We have strengthened the review’s rationale by adding statistics and data related to adverse services and reproductive health problems of women with physical disabilities. These updates are included in the Introduction (please see lines 61-82). |
| 4 | Inclusion criteria ambiguity: Mention of including studies with design flaws may lead to inconsistencies in data synthesis. Clearer inclusion/exclusion criteria are required for reliability |
|  | Thank you for your feedback. we have revised the inclusion and exclusion criteria to provide clearer and more detailed guidelines. These updates can be found in the Methods section (please see line 166-184). |
| 5 | Stakeholder consultation details: While the protocol mentions consulting stakeholders, it does not detail how these consultations will be conducted or integrated into the review process. |
|  | We appreciate your suggestion to provide more details on how these consultations will be conducted and integrated into the review process. In response, we have added a detailed description of the stakeholder consultation process (please see line 248-255). |
| 6 | Lack of preliminary findings: Although it is a protocol, including a section outlining expected outcomes based on preliminary literature could provide context and significance to the review. |
|  | Thank you for your valuable suggestion regarding the inclusion of preliminary findings. As we are currently in the screening phase, we have incorporated the preliminary findings from the study search into the manuscript. (Please see line 256-259). |
| 7 | Reporting framework: The document states results will follow the PRISMA-ScR checklist but does not specify how findings will be categorized or organized |
|  | Thank you for your feedback. We have clarified the categorization of findings according to the PRISMA-ScR checklist. Results will be organized into:  1. Reproductive health programs,  2. Reproductive health status,  3. Reproductive health needs, and  4. Reproductive health challenges for women with physical disabilities.  These categories will ensure a structured and comprehensive presentation of the findings, aligning with the objectives of the review. We have updated the Methods section to reflect this organization (please see lines 234-237). |
| 8 | Limited engagement with existing literature: The discussion could better engage with existing studies to contextualize the identified gaps in the literature |
|  | We sincerely appreciate your valuable suggestion. We fully agree that greater engagement with existing studies can enhance the understanding of gaps in the literature. In response to your feedback, we have revised the Discussion section by incorporating additional references (please see the Discussion section, line 266-281). |
| 9 | Future research directions: While mentioning that the review will guide future research, the document does not specify potential areas for exploration, leaving actionable insights for researchers vague. |
|  | We are deeply grateful for your insightful feedback. we have enriched the Discussion section by incorporating additional content aimed at resolving the identified issues (please see the Discussion section, line 266-295 ). |
| 10 | Clarity and grammatical issues: Various grammar, sentence structure, and language clarity issues need addressing for better readability and professionalism |
|  | Thank you for your feedback on grammar and clarity issues. We have revised the manuscript to improve sentence structure, grammar, and readability and utilized the services of a professional language editor to address grammatical and typographical errors.  We appreciate your time and welcome any further suggestions. |

We have incorporated all the suggested revisions and hope that the revised manuscript now meets the journal's standards. Should you require any further clarification or additional information, please do not hesitate to contact us.

Once again, we sincerely thank you and the reviewers for your time and valuable input. We look forward to hearing from you regarding the next steps.

Best regards,
Maryam Abedi
m.abedi931@gmail.com
2025.1.14
