# Supplementary material for: Reproductive health programs in women with physical disabilities: A scoping review protocol
Source: PLoS One. 2025 Aug 6;20(8):e0329124. doi: 10.1371/journal.pone.0329124 (PMC12327595; doi:10.1371/journal.pone.0329124)
Supplement: Attachment — Submitted filename: Response_to_Reviewers_auresp_2.docx [file pone.0329124.s006.docx]

**Subject:** Response to Reviewer Comments on the Manuscript titled " **Reproductive health programs in women with physical disabilities: a scoping review protocol"**

**Dear Editor and Reviewer,**

Thank you sincerely for your time, expertise, and thoughtful feedback on our manuscript titled"**Reproductive health programs in women with physical disabilities: a scoping review protocol".** We deeply appreciate your constructive critiques and actionable suggestions, which have significantly strengthened the rigor and clarity of our work.

In response to your comments:
We have carefully considered each point and incorporated revisions to address them comprehensively. Below, we provide a detailed, point-by-point response to your feedback, along with the specific changes made in the revised manuscript (highlighted in track changes or noted by line/page numbers).

| 1 | The research gap still not clear. |
| --- | --- |
| answer | Thank you for your careful attention to this important point. In the revised introduction section (lines 89-107), we have clarified the research gap by adding examples of key limitations, including:   - Lack of evidence on interventions to improve access to reproductive health services for women with physical disabilities in underprivileged areas, - Dominant focus of existing studies on challenges and barriers faced by women, without addressing health promotion programs, - Scarcity of longitudinal studies to assess the sustainability and long-term impact of interventions, - Inequity in existing data, with a predominance of evidence from high-income regions and neglect of rural/remote communities.   We hope these revisions more accurately reflect unaddressed research gaps in the reproductive health of women with physical disabilities. We sincerely appreciate your insightful feedback and welcome any additional suggestions to further improve this work. |
| 2 | The authors have chosen to retain the term "disabled" rather than using "individuals with special needs." While this is justified, the manuscript should consistently use person-first language (e.g., "women with disabilities" rather than "disabled women") |
| answer | We sincerely appreciate the reviewer’s insightful feedback regarding the use of person-first language. We fully align with the importance of emphasizing individuality and dignity by using terms like “**women with disabilities**” instead of “**disabled women**”. In response to this guidance, we have meticulously revised the entire manuscript to ensure consistent application of person-first language.  Thank you for this valuable suggestion, which has strengthened the manuscript’s inclusivity and precision. |
| 3 | The authors mention using the Joanna Briggs Institute (JBI) Quality Appraisal tool but do not specify how the results of this assessment will be used. Will low-quality studies be excluded, or will the quality assessment simply inform the interpretation of findings? This needs to be clarified. |
| answer | Thank you for your insightful comments. As the purpose of this scoping review is to map existing reproductive health programs for women with disabilities, our primary goal is not to draw conclusions or identify effective programs or select studies based on quality or exclude low-quality studies. Our goal is simply to:   - Provide a comprehensive overview of the existing evidence quality and methodological characteristics of the included research, - Identify critical research gaps in the field, and - Aid in the interpretation of findings while accounting for potential study limitations.   The results of this assessment will be fully included in the final article.  Thank you again for this important point, which greatly contributed to the article's clarity.  Please see the changes related to this section on lines 221-227. |
| 4 | Some sections of the manuscript, particularly the methods, are still difficult to follow due to overly complex sentence structures. Also, some minor language defects, I suggest proof reading by a native English. |
| answer | Thank you for your meticulous feedback on improving the manuscript’s clarity and language quality. We deeply value your expertise and have implemented the following revisions to address your concerns:   - Complex sentences in the Methods section (e.g., search strategy, inclusion criteria) have been restructured into shorter, step-by-step descriptions - The entire manuscript underwent rigorous grammatical and stylistic revisions to resolve ambiguities and improve flow. - A native English-speaking editor (certified by ***NativeEnglishEdit.com***, with REFER CODE:EE-2024-333326096- ABEDI-2870 NEW 1122 REV.2) proofread the final version to ensure linguistic precision and natural phrasing.   We believe these revisions align the manuscript with the journal’s standards for clarity and rigor. Should any sections still require refinement, we are fully committed to further adjustments.  Thank you again for your invaluable guidance in strengthening this work. |
| 5 | how stakeholders will be selected and how their input will be integrated. |
| answer | We sincerely appreciate the reviewer’s insightful comment regarding stakeholder engagement. In response, we have elaborated on both the stakeholder selection process and the strategies for integrating their input in the revised manuscript. These details are now included in the section titled *"Consulting with Relevant Stakeholders"* (Lines 244–264).  Thank you again for your thoughtful feedback, which helped us improve the clarity and completeness of this section. |

We thank you and the reviewer for your thorough assessment. All comments have been addressed, and necessary revisions implemented. I hope the revised manuscript meets the journal's standards.

 We deeply appreciate your time and guidance and eagerly await your response.

Best regards,

Zahra Motaghi

[zhrmotaghi1@gmail.com](mailto:zhrmotaghi1@gmail.com)

Shahroud University of Medical Sciences and Health Services, Shahroud, Iran
